# Supplementary material for: Subtle differences in the pathogenicity of SARS-CoV-2 variants of concern B.1.1.7 and B.1.351 in rhesus macaques
Source: Sci Adv. 2021 Oct 22;7(43):eabj3627. doi: 10.1126/sciadv.abj3627 (PMC8535829; doi:10.1126/sciadv.abj3627)
Supplement: Supplementary file 1 — Figs. S1 to S7 Tables S1 and S2 [file sciadv.abj3627_sm.pdf]

Science Advances
AAAS

Supplementary Materials for

Subtle differences in the pathogenicity of SARS-CoV-2 variants of concern B.1.1.7 and B.1.351 in rhesus macaques

Vincent J. Munster, Meaghan Flagg, Manmeet Singh, Claude Kwe Yinda, Brandi N. Williamson, Friederike Feldmann, Lizzette Pérez-Pérez, Jonathan Schulz, Beniah Brumbaugh, Myndi G. Holbrook, Danielle R. Adney, Atsushi Okumura, Patrick W. Hanley, Brian J. Smith, Jamie Lovaglio, Sarah L. Anzick, Craig Martens, Neeltje van Doremalen, Greg Saturday, Emmie de Wit*

*Corresponding author. Email: emmie.dewit@nih.gov

Published 22 October 2021, *Sci. Adv.* **7**, eabj3627 (2021)
DOI: 10.1126/sciadv.abj3627

This PDF file includes:

Figs. S1 to S7
Tables S1 and S2

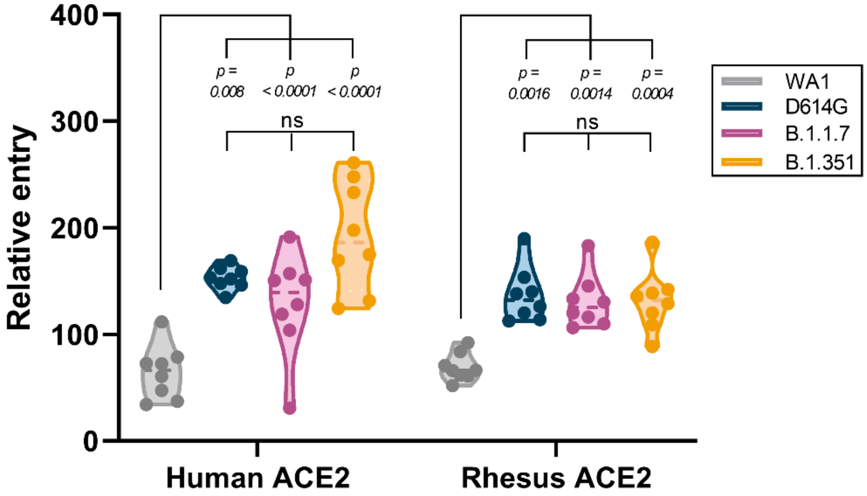

Figure S1. Comparison of binding of SARS-CoV-2 spike proteins to human and rhesus macaque ACE2. BHK cells expressing either human ACE2 or rhesus ACE2 were infected with pseudotyped VSV reporter particles with the spike proteins of WA1, D614G, B.1.1.7 and B.1.351, luciferase was measured and normalized to no spike controls as a readout for cell entry. The data are representative of eight replicates; dotted line indicates the mean. Statistical analysis was performed using a 1-way ANOVA with Sidak's multiple comparisons test.

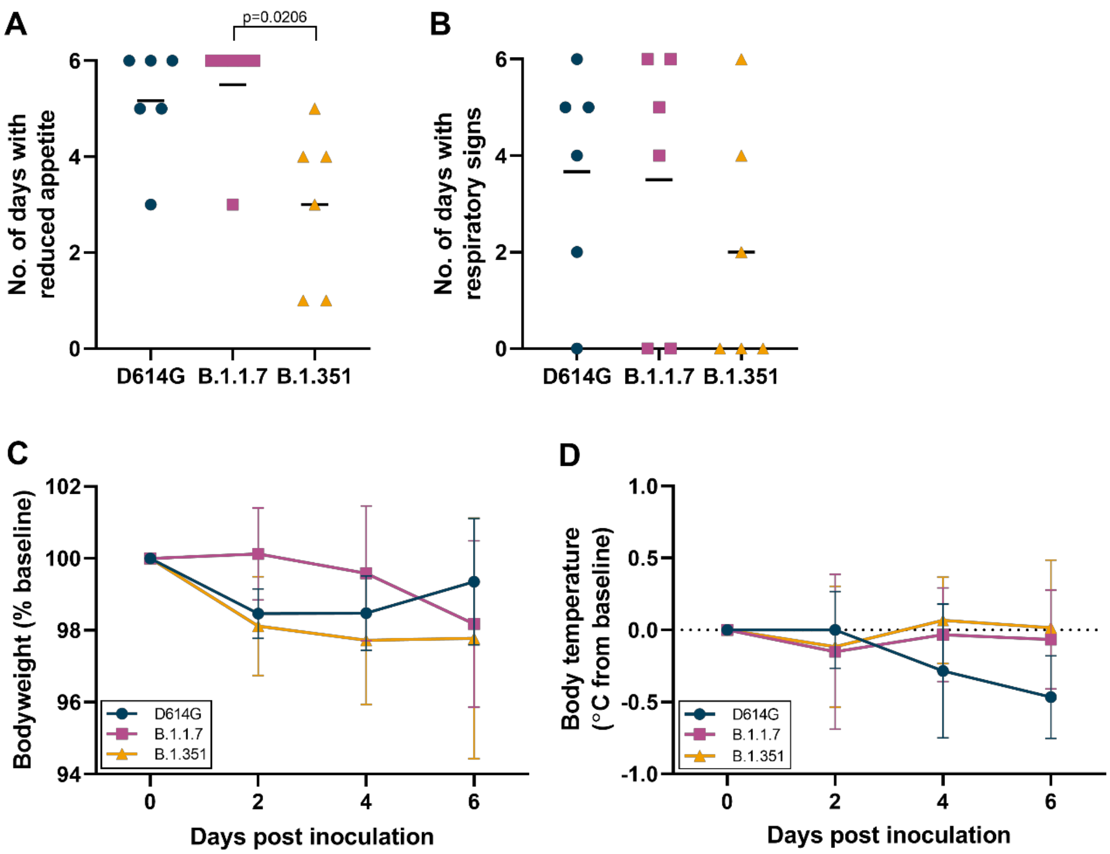

Figure S2. Clinical disease observed in rhesus macaques inoculated with D614G, B.1.1.7 and B.1.351. Three groups of six adult rhesus macaques were inoculated with SARS-CoV-2 variants D614G, B.1.1.7 or B.1.351. After inoculation, animals were observed for disease signs and scored according to a pre-established clinical scoring sheet. The number of days an animal showed reduced appetite (A) or changes in respiration pattern (B) are indicated. On exam days, bodyweight (C) and body temperature (D) were measured and plotted as changes from baseline. Statistical analyses were performed using a Kruskal-Wallis test with Dunn's multiple comparisons (A, B) or a 2-way ANOVA with Tukey's multiple comparisons test (C, D); p-values <0.05 are indicated.

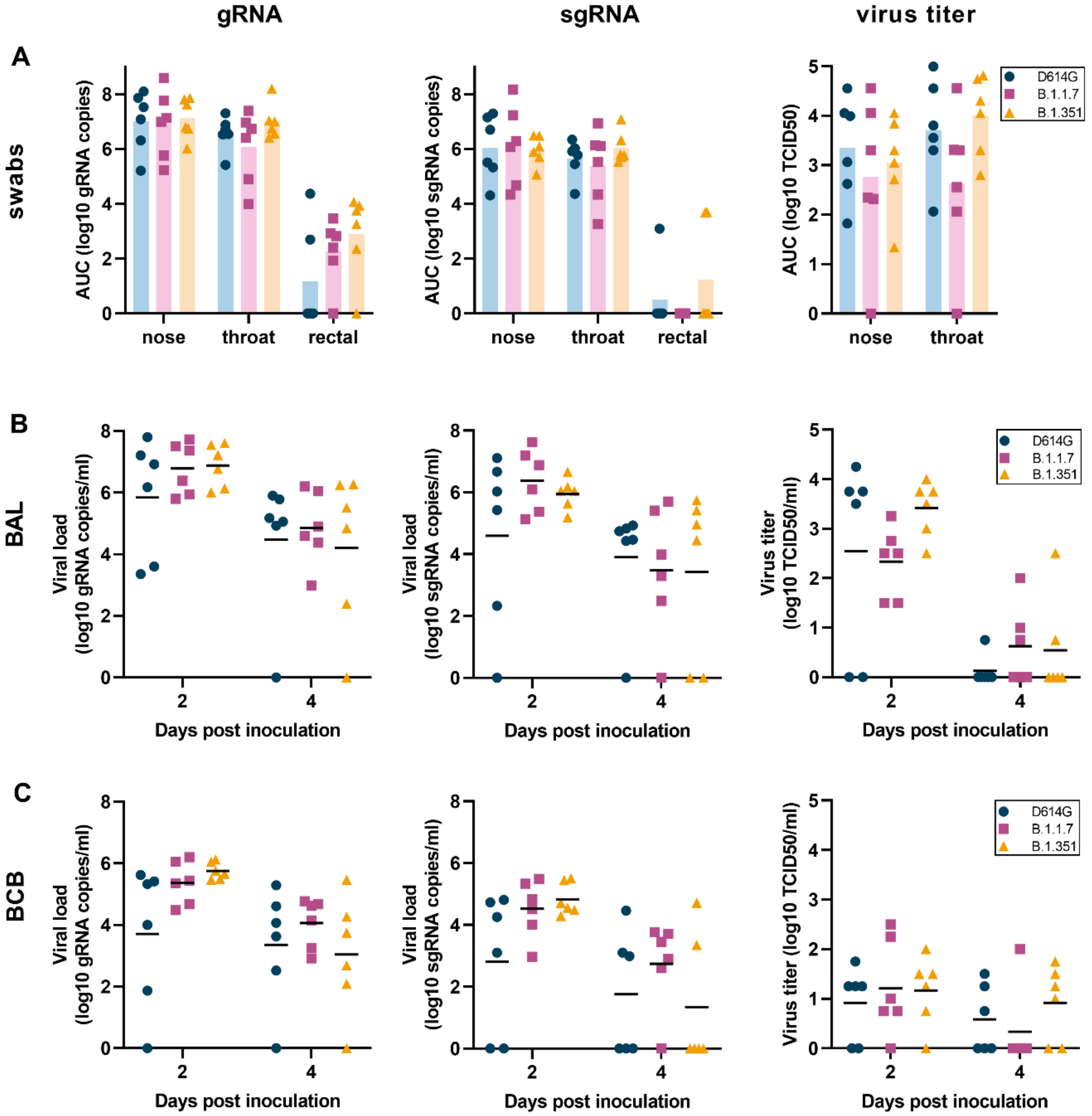

Figure S3. SARS-CoV-2 viral loads and virus titers in swabs, bronchoalveolar lavages and bronchial cytology brushes. Three groups of six adult rhesus macaques were inoculated with SARS-CoV-2 variants D614G, B.1.1.7 or B.1.351. After inoculation, clinical exams were performed on 2, 4, and 6 dpi during which nose, throat, and rectal swabs were collected. qRT-PCR was performed to detect genomic (left column) and subgenomic RNA (middle column), and virus titration was performed to detect levels of infectious virus (right column) and the Area Under the Curve (AUC) was calculated as an indication of the total amount of virus shed in these samples. Swabs (a), bronchoalveolar lavages (BAL) (b) and bronchial cytology brush (BCB) samples (c) were collected on 2 and 4 dpi and analyzed for the presence of gRNA, sgRNA and infectious virus. Lines indicate the mean. Bars (a) and lines (b, c) indicate the mean. Statistical analysis was performed using a Kruskal-Wallis test with Dunn's multiple comparisons tests (a) or a 2-way ANOVA with Tukey's multiple comparisons test (b, c); no p-values <0.05 were found.

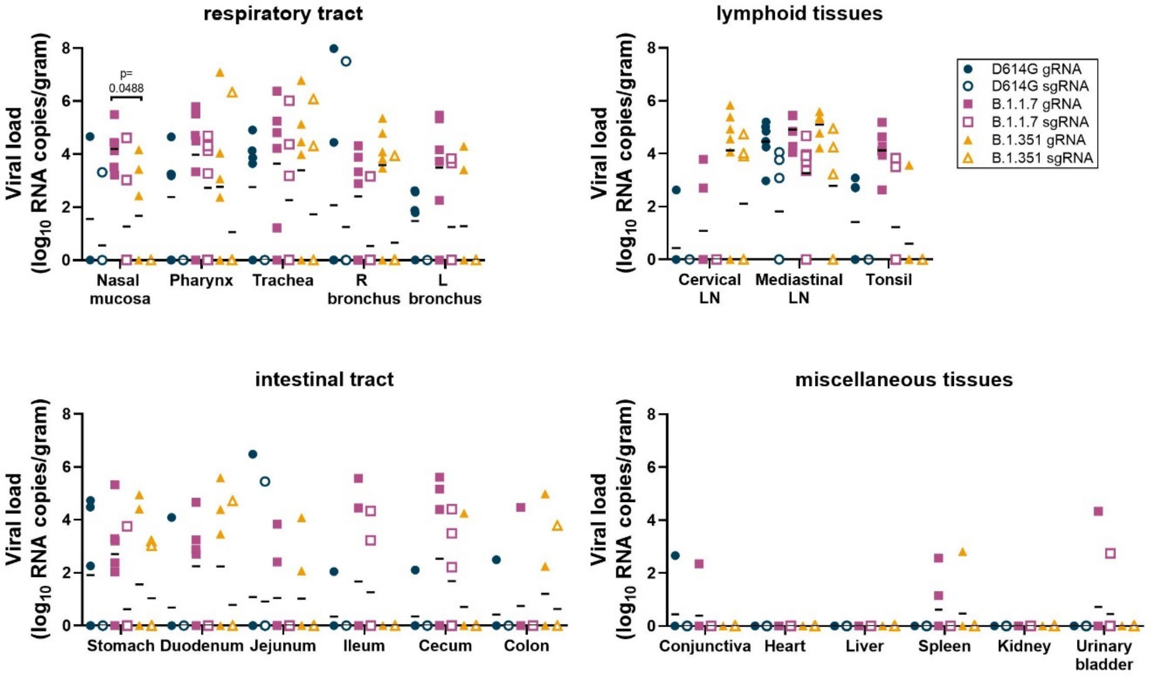

Figure S4. Viral loads in tissues collected from D614G, B.1.1.7 or B.1.351-inoculated rhesus macaques on 6 dpi. Three groups of six adult rhesus macaques were inoculated with SARS-CoV-2 variants D614G, B.1.1.7 or B.1.351. On 6 dpi, all animals were euthanized and necropsies were performed. Samples were collected from many different organs and analyzed for the presence of gRNA (closed symbols) and sgRNA (open symbols). Lines indicate the mean. Statistical analysis was performed using a 2-way ANOVA with Tukey's multiple comparisons test; p-values <0.05 are indicated.

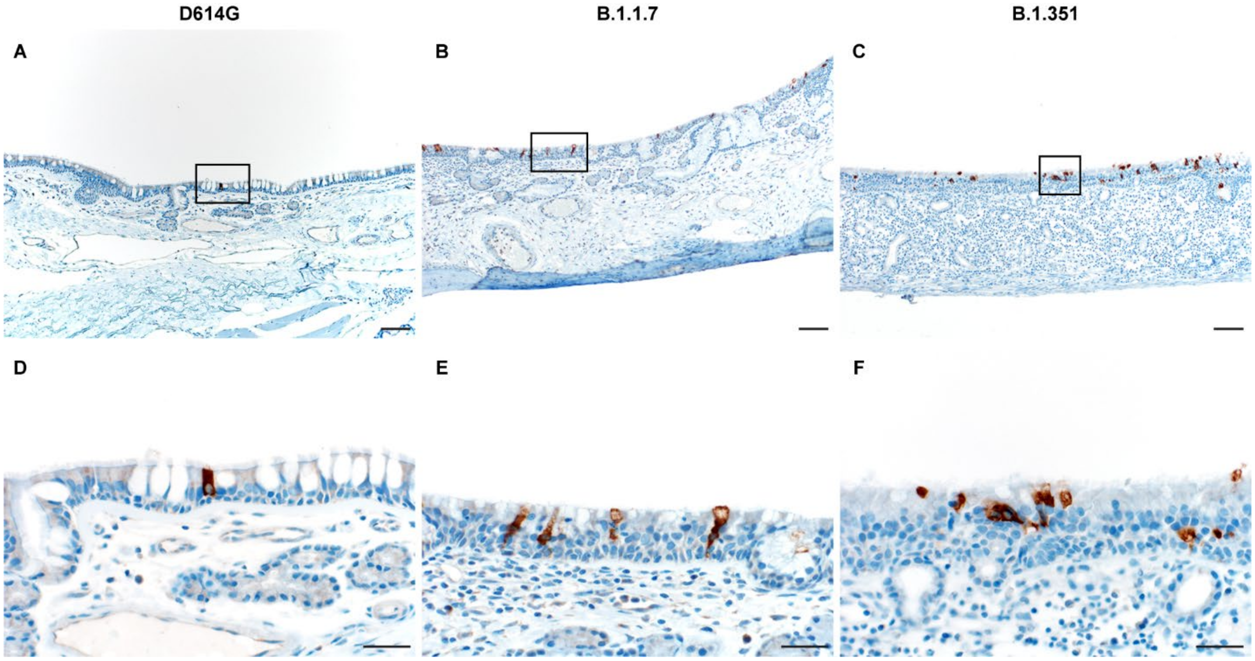

Figure S5. Viral antigen in the nasal epithelium of rhesus macaques inoculated with a D614G, B.1.1.7 or B.1.351 isolate of SARS-CoV-2. The Three groups of six adult rhesus macaques were inoculated with SARS-CoV-2 variants D614G, B.1.1.7 or B.1.351. On 6 dpi, all animals were euthanized and necropsies were performed. Nasal turbinates were assessed for the presence of SARS-CoV-2 antigen in D614G (A, D), B.1.1.7 (B, E) or B.1.351 (C, F) inoculated animals. D, E, F are close-ups of the noxed areas in A, B, and C, respectively. Scale bars: 50µm (A-C) or 20µm (D-F).

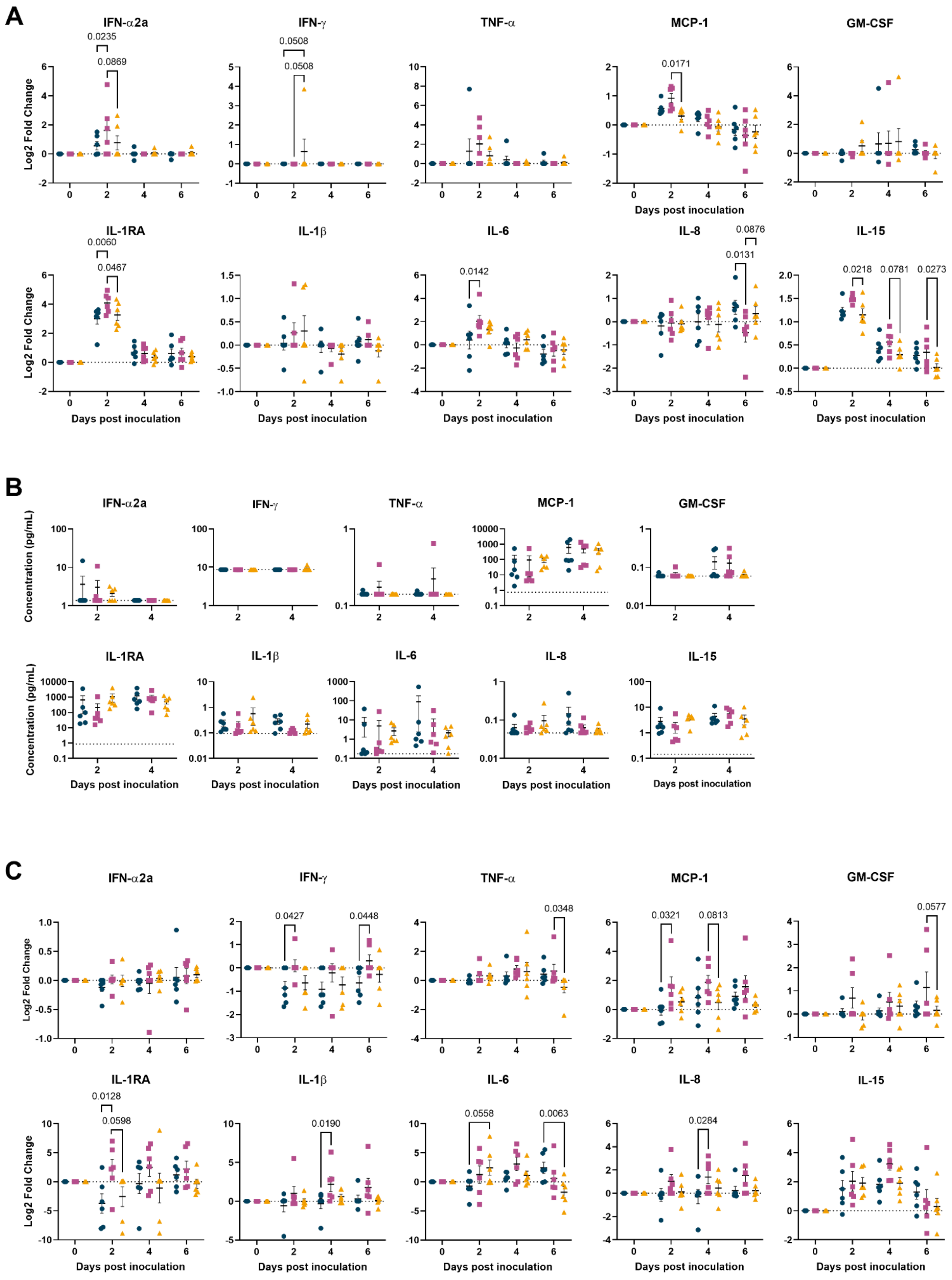

A
IFN-α2a
IFN-γ
TNF-α
MCP-1
GM-CSF
IL-1RA
IL-1β
IL-6
IL-8
IL-15
Log2 Fold Change
Days post inoculation
0.0235
0.0869
0.0508
0.0508
0.0171
0.0060
0.0467
0.0142
0.0876
0.0131
0.0218
0.0781
0.0273
B
Concentration (pg/mL)
C
0.0427
0.0448
0.0348
0.0321
0.0813
0.0577
0.0128
0.0598
0.0190
0.0558
0.0063
0.0284

Figure S6. Cytokine and chemokine responses in rhesus macaques inoculated with a D614G, B.1.1.7 or B.1.351 isolate of SARS-CoV-2. The concentration of 10 different cytokines and chemokines were measured in serum (a), BAL (b), and nasal samples (c) at different timepoints before and after inoculation. Fold 2 log changes were calculated for samples where baseline (0 dpi) values were available (a, c); in the absence of a baseline sample concentrations are plotted in pg/mL (b). Blue circles: D614G; pink squares: B.1.1.7; yellow triangles: B.1.351. Dotted lines indicate no change from baseline (a, c) or the maximum lower limit of detection calculated across plates (b). Statistical analysis was performed using a 2-way ANOVA with Tukey's multiple comparisons test; p-values <0.05 are indicated.

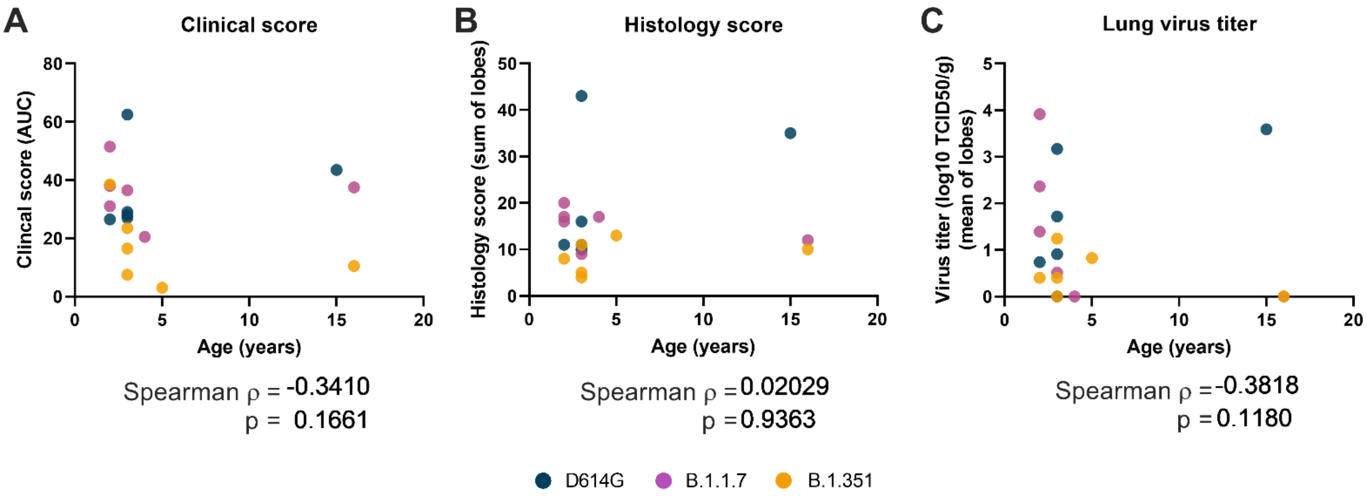

Figure S7. Clinical score, histology score, and lung virus titers are not associated with age. As a measure of total disease severity, we calculated the area under the curve (AUC) of clinical scores for each animal over six days of observation. We tested clinical score AUC (A), histology score (sum of scores for each lung lobe; B), and lung virus titer (average of titers for each lung lobe; C) for association with animal age using Spearman rank correlation. Spearman's correlation coefficient (ρ) and p-value are indicated below each graph.

Table S1. Genome changes observed in virus inoculum and 2dpi BAL samples as compared to reference consensus sequence as reported in GenBank (D614G: MW127503.1) or GISAID (GISAID: B.1.1.7: EPI_ISL_683466; B.1.351: EPI_ISL_890360). Changes are indicated in allelic fraction calculated after filtering as indicated in Methods.

					Animal no.			
**ORF**	**a.a. change**	**inoculum**	**RM1**	**RM2**	**RM3***	**RM4**	**RM5**	**RM6**
**D614G**								
nsp6	H3631H	<0.1	<0.1	0.98	0	<0.1	<0.1	<0.1
**B.1.1.7**		**inoculum**	**RM7**	**RM8**	**RM9**	**RM10**	**RM11**	**RM12**
nsp6	D165G	0.14	<0.1	<0.1	0.16	<0.1	<0.1	<0.1
nsp6	F181V	<0.1	<0.1	<0.1	<0.1	<0.1	0.10	<0.1
nsp6	L257F	0.18	0.22	0.23	0.28	0.22	0.20	0.18
nsp7	V11I	0.13	0.22	0.21	<0.1	0.28	0.21	0.45
**B.1.351**		**inoculum**	**RM13**	**RM14****	**RM15**	**RM16**	**RM17**	**RM18**
nsp5	P252L	0.17	0.32	<0.1	0.31	0.28	0.34	0.30
nsp6	L257F	0.57	0.47	0.64	0.51	0.58	0.48	0.53

*Poor read coverage

**Marginal read coverage

Table S2. Age of rhesus macaques used in this study.

D614G		B.1.1.7		B.1.351	
Animal no.	Age (years)	Animal no.	Age (years)	Animal no.	Age (years)
RM1	15	RM7	16	RM13	16
RM2	3	RM8	4	RM14	5
RM3	3	RM9	2	RM15	3
RM4	3	RM10	2	RM16	3
RM5	2	RM11	3	RM17	3
RM6	3	RM12	2	RM18	2
